# Predictive Value of the Fear-Avoidance Model on Functional Capacity Evaluation

**DOI:** 10.1007/s10926-017-9737-7

**Published:** 2017-11-01

**Authors:** Johanne Tüscher, Cyrille Burrus, Philippe Vuistiner, Bertrand Léger, Gilles Rivier, François Luthi

**Affiliations:** 1 Faculty of Biology and Medicine, University of Lausanne, Rue du Bugnon 21, 1011 Lausanne, Switzerland; 2 Department for Musculoskeletal Rehabilitation, Clinique Romande de Réadaptation Suvacare, Avenue Grand-Champsec 90, 1950 Sion, Switzerland; 3 Institute for Research in Rehabilitation, Clinique Romande de Réadaptation Suvacare, Avenue Grand-Champsec 90, 1950 Sion, Switzerland; 4 Department of Physical Medicine and Rehabilitation, Orthopaedic Hospital, Lausanne University Hospital, Rue du Bugnon 21, 1011 Lausanne, Switzerland

**Keywords:** Fear-avoidance model, Functional capacity evaluation, Physical performance

## Abstract

*Purpose* Measuring the predictive value of the Fear-Avoidance Model (FAM) on lifting tasks in Functional Capacity Evaluation (FCE), and on reasons for stopping the evaluation (safe maximal effort, versus self-limited). *Methods* A monocentric prospective study was conducted on 298 consecutive inpatients. Components of the FAM were analyzed using the Cumulative Psychosocial Factor Index (CPFI: kinesiophobia, catastrophizing, depressive mood) and perceived disability (Hand/Spinal Function Sort: HFS/SFS). Floor-to-waist, waist-to-overhead and dominant-hand lifting tests were measured according to the FCE guidelines. Maximal safe performance was judged by certified FCE assessors. Analyses were conducted with linear multiple regression models. *Results* The CPFI was significantly associated with the 3 FCE lifting tests: floor-to-waist (ß = − 1.12; p = 0.039), waist-to-overhead (ß = − 0.88; p = 0.011), and dominant-handed lifting (ß = − 1.21; p = 0.027). Higher perceived disability was also related to lower performances: floor-to-waist (ß = 0.09; p < 0.001), waist-to-overhead (ß = 0.04; p < 0.001), and dominant-handed lifting (ß = 0.06; p < 0.001). The CPFI was not related to performances of patients with self-limited effort despite higher psychological scores, while a relationship was found for patients who achieved a safe maximal performance. Higher perceived disability was related to performances in both situations. *Conclusions* FAM components should be taken into account when interpreting maximal physical performance in FCE. This study also suggests that factors other than pain-related fears may influence patients with self-limited effort.

**Electronic supplementary material:**

The online version of this article (doi:10.1007/s10926-017-9737-7) contains supplementary material, which is available to authorized users.

## Introduction

The Fear-Avoidance Model (FAM) has been used for more than 20 years and has gained in popularity as a theoretical model explaining development and perpetuation of chronic pain. It describes how physical disuse may develop as a result of persistent avoidance behaviors influenced by fear beliefs of movement or pain, misinterpretations of pain, and affective distress [1]. Even if the FAM is widely accepted as an explicative model, there is still need to understand interactions between its dimensions and its predictive influence on chronic disability [1–3]. Besides, the influence of the FAM is mainly based on subjective assessment [2, 4]. There are few studies addressing relationships of the FAM with more objective outcomes, and they are mainly made in laboratory conditions. For instance, the FAM was related to isokinetic strength assessment [5], static [6] or repetitive lifting of a bag [7], and walking speed [8]. Therefore, assessment of the FAM role on functional tasks in clinical and ecological environments is needed [1, 2].

In clinical settings, it might be difficult to transpose standardized and properly controlled conditions used in laboratory testing. The level of maximal physical performance achieved by chronic pain patients may be hard to determine [9] and may influence the relationships between predictors and outcomes. In other words, it is difficult to distinguish between a maximal effort or a self-limited effort. Functional Capacity Evaluations (FCE) are often used in vocational rehabilitation to assess work-related physical performance. They are based on standardized measurements, with a clear definition of a safe maximal performance, and have been validated in the last decades [10–12]. Self-efficacy and various biological factors such as age or gender are known to influence FCE performances [13–16]. The relationship between the FAM and the FCE is still debated and studies have produced mixed results [16], mainly due to various settings, different available tools to assess the FAM and lack of meaningful clinical cut-off scores.

The cyclical relationship of the FAM is also under debate [2, 17, 18] and close relationships between the different components of the FAM induce collinearity which adds difficulties to determine associations between the FCE and each individual FAM component in regression analyses [19]. In an attempt to improve the clinical utility of the FAM and its applicability for medical decision-making, Wideman et al. have proposed a cumulative interpretation and developed the Cumulative Psychosocial Factor Index (CPFI) using cut-off scores for the FAM psychological components [20]. The CPFI is valuable to reduce the aforementioned difficulties and is applicable to all groups of patients with musculoskeletal pain. It was found to be predictive of pain persistence and work disability [20]. To date, it has never been used as a cumulative risk index measuring the influence of the FAM on physical performances.

The aim of our study was to investigate the influence of the FAM on FCE performances by using the CPFI and to answer the following questions: (1) What is the predictive value of the FAM on FCE lifting performances in patients with chronic musculoskeletal pain? (2) What is the contributing role of the FAM on reasons for stopping lifting tests? Our hypotheses were that the FAM would influence the performance during FCE lifting tests and that it would have a greater influence in patients with self-limited effort.

## Methods

### Study Design

We conducted a prospective study at the Clinique Romande de Réadaptation, a tertiary rehabilitation center in the French-speaking part of Switzerland.

### Participants

Any patient of working age (18–65 years old) who was referred to our clinic after an accident for a rehabilitation program with a vocational aspect and had an FCE was eligible for the study. Exclusion criteria were patients with spinal cord or traumatic brain injuries, incapable of judgement or under legal custody. Patients were referred from all French-speaking counties of Switzerland, including urban and industrial city centres or more rural regions by general practitioners, surgeons or insurance medical advisors when they presented chronic musculoskeletal pain (≥ 3 months), functional impairments and inability to return to work after orthopaedic injuries following work, traffic, sport or leisure activities. Health and accident insurances are compulsory in Switzerland and patients are insured against occupational and non-occupational injuries. The insurer must pay for medical treatment as long as substantial improvement can be anticipated. The insured persons have a legal right to integration measures, but they are obliged to cooperate and do everything possible to return to an occupational activity, avoiding the need for pension. Rehabilitation initially takes place close to patients’ residence. They are sent to a tertiary rehabilitation center when the evolution is unfavourable or they cannot return to their regular occupation. The aim of the therapeutic program was to manage patients using a multidisciplinary biopsychosocial approach according to the practice recommendations for chronic pain patients [21]. This program involves physical components (physiotherapy and occupational therapy with individual and group sessions, including graded exercises and functional training), vocational training, social advice and psychological components with a cognitive approach. The length of stay is 4–5 weeks with at least 3–4 h of daily therapies (excluding weekends). The inclusion period ran from May 2013 to September 2015. If a patient was treated twice during this period, we considered only data from the first hospitalization. Patients gave an oral informed consent. The study was conducted according to the principles expressed in the ‘‘Declaration of Helsinki 2008’’ and the protocol was approved by the local medical ethics committee (CCVEM 034/12).

### Measurements

#### Patients’ Background

Patients’ characteristics and socio-demographic data were selected for their potential confounding influence on the FCE performances [16] and were collected at the admission from medical assessment and divided into biological and social factors. Biological variables included were (1) age; (2) gender; (3) body mass index (BMI); (4) injury location (upper limb, lower limb, spinal, or multiple sites injuries); (5) Abbreviated Injury Scale (AIS) score (minor injury versus moderate or serious injury) [22]; (6) interval between injury and hospitalization in days; and (7) Brief Pain Inventory (BPI) severity subscale, which assesses pain severity (range, 0–10) [23, 24]. Social variables consisted of (1) native language (French versus other); (2) education level (high: ≥ 9 years compulsory schooling and/or qualified work versus low: ≤ 9 years and unqualified work); (3) employment contract (yes versus no); and (4) work related injury (yes versus no).

#### Cumulative Psychological Factor Index (CPFI)

**Fig. 1:**
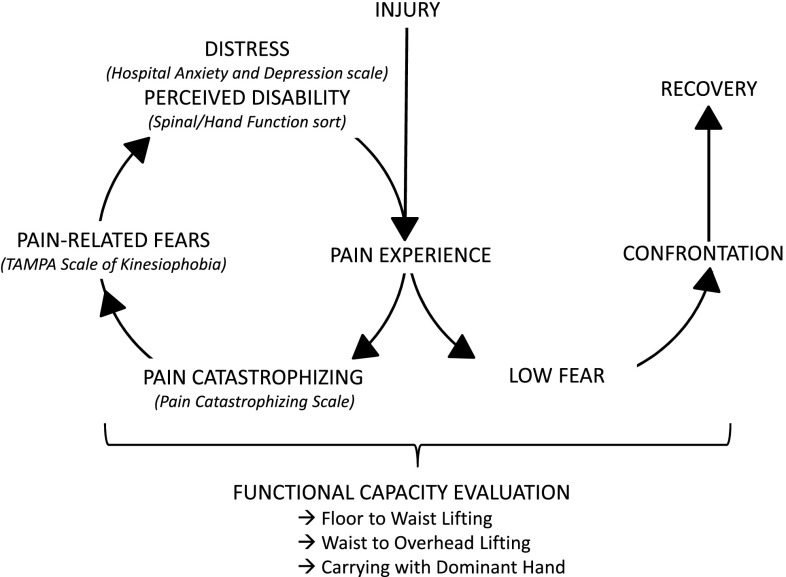
Illustration of the full FAM assessment

The FAM components were identified as potential predictors and were assessed using data collected from standard reference self-reported questionnaires answered during the first two days after admission. All questionnaires were available in previously translated and validated versions (French and several foreign languages) and are detailed further in the paragraph. In order to investigate the potential cumulative effect of elevated FAM psychological dimensions on FCE performances during lifting tasks, we used the Cumulative Psychological Factor Index (CPFI) as described by Wideman and Sullivan [20]. To complete the components of the FAM, perceived disability was also treated as a potential predictor of the outcomes (see Fig. 1). The CPFI includes 3 psychological dimensions of the FAM assessed with the following questionnaires: (1) the Pain Catastrophizing Scale PCS) which consists of 13 questions measuring catastrophic thoughts related to pain (range 0–52), higher score suggesting catastrophizing [25]. The clinical established CPFI cut-off for the PCS is ≥ 20/52, meaning that for a patient with a score ≥ 20 points poorer outcomes are expected [20]; (2) the Tampa Scale for Kinesiophobia (TSK) which assesses fear of movement/(re)injury using a 17 item scale (range 17–68), higher score suggesting high kinesiophobia [26]. In order to improve the selection of patients with high kinesiophobia, we used a clinical cut-off of ≥ 45/68 for the TSK, according to the norming data suggested by Roelofs [27], which is slightly different from the original cut-off established by Wideman and Sullivan (≥ 38/68) [20]; (3) As the Beck Depression Inventory (BDI), which was used in the original CPFI version to assess depressive symptoms [20], was not available in our clinic, we used the depression subscale of the Hospital Anxiety and Depression Scale (HAD-D), another standard reference questionnaire [28]. The depressive subscale contains seven questions (range 0–21), a higher score suggesting higher depressive symptoms. The cut-off score for the HAD-D is ≥ 8/21 [28]. The CPFI represents the number of scores above the given cut-offs, i.e. ranging from 0 (below the cut-off for all scales) to 3 (above or equal to the cut-off for all three scales).

#### Hand Function Sort (HFS) and the Spinal Function Sort (SFS)

The perceived disability was measured by the Hand Function Sort (HFS) and the Spinal Function Sort (SFS), which are pictorial questionnaires assessing perceived capacity to achieve physical activities related to work and daily activities, including material handling, postural tolerance and ambulation. The HFS (62 tasks; range 0–248) was used for patients with upper limb injuries [29] and the SFS (50 tasks; range 0–200) for patients with spinal or lower limb injuries [30]. In order to be able to compare the scores, we rescaled the HFS score to a maximum of 200 points.

### Outcomes

We focused on the three lifting tests of the FCE: the floor-to-waist test, the waist-to-overhead test and the dominant-handed carrying test. The FCE tests were performed at the end of the hospitalization according to Isernhagen FCE standards [12]. These tests are used in FCE as they present routine tasks for manual workers. With other tests, they help determine the individual’s functional capacity and if it matches with job physical demands that are obtained from the Dictionary of Occupational Titles [31]. Patients were instructed on how to perform the tests and were asked to give their maximal effort. Assessors (physiotherapists) were all FCE certified and regularly trained and were different from the attending physiotherapist of rehabilitation in order to minimize the risk of bias due to interactions between assessors and participants. They were also blinded for the CPFI score. Maximal performances are obtained in approximately 5 consecutive steps with gradual increments of 2.5 or 5 kg, the load added relying on FCE assessors’ judgement. To validate a step, the load must be lifted five times for the floor-to-waist and the waist-to-overhead tests and carried over a distance of 15 m for the dominant-handed carrying test within 90 s, before the assessor increases the weight. Maximal safe performance is judged by the FCE assessor who uses the following criteria based on biomechanical and physiological signs in response to the effort: bulging of prime movers and accessory muscles, very wide base, counterbalance, increase in heart and respiration rate, very slow pace and safe lifting but inability to maintain control with the addition of any more weight [32]. Unless maximal performance is reached, the test may be interrupted for safety reasons by the assessor or by the patient himself if he feels he cannot perform better. In the latter case, the performance is considered as self-limited [9].

### Data Collection and Bias

To minimize the measurement bias, questionnaires and other clinical and demographic data were collected before starting the therapeutic program. Records were collected with a digital pen, which permits data capture and direct transfer from paper to the data files. Questionnaires were administered in the 2–3 days following entry. Functional capacity evaluation were performed 3–4 weeks after admission and were done under the supervision of highly experienced physiotherapists, familiar with the tests through regular training and clear instructions.

### Data Analysis

To study the association between the FAM and the FCE lifting tests, we used a Cumulative Psychological Factor Index (CPFI) as suggested by Wideman and Sullivan [20]. Scores of the PCS, TSK and HAD-D were split according to clinically established cut-offs (20, 45, and 8, respectively [20, 27, 28]).

The three FCE tests selected were not available for all patients. The entire cohort underwent the floor-to-waist lift, 281 patients performed the waist-to-overhead lift and 283 the dominant-handed carrying test. A total of 86 individuals (28.9%) had missing information in one or the other variable. TSK and HAD-D questionnaires were filled out by 269 patients, PCS by 256 patients while the HFS/SFS scores were available for 276 patients. Nevertheless, no single variable presented more than 15% missing observations, the highest proportion being in the BPI severity score (43/298, i.e. 14.4%).

The probability that data are missing is known to depend on some biopsychosocial factors [33], thus data are not missing completely at random (MCAR), i.e. the 212 complete-case patients (representing 71.1% of the sample) do not represent a random sample of the whole data. Simply discarding the missing values would result in potential bias, in addition to losing statistical power. Nevertheless, the factors known to be associated with nonresponse are all measured and taken into account in the statistical models, that is why the assumption of missing at random (MAR) is plausible, i.e. the probability of data being missing does not depend on unobserved data, given the observed data. In this situation, multiple imputation is likely to improve the measures of associations compared to complete-case analysis [34].

Multiple imputations by chained equations (MICE) were applied [35]. Continuous variables were imputed using linear regression models and binary variables, with logistic regression models. Normality was assumed for all continuous variables except the interval between injury and hospitalization, for which a log-transformation was applied prior to the imputation. The number of imputed data sets should be at least equal to the percentage of incomplete cases [35], thus we used 30 for the analyses. A sensitivity analysis was performed on complete-case data (N = 212).

Multiple linear regression models were applied to measure the associations between FCE tests and the CPFI and HFS/SFS scores, while adjusting these associations for potential confounders. The available sample size allowed the estimation of up to 19 parameters in regression models to keep a minimum of 15 observations per parameter [36].

In a second step, we compared patients who performed a safe maximal effort and those who did not (self-limited) for each FCE test separately. The same regression models as above were performed, further including this binary variable and its interaction with the CPFI and with HFS/SFS score as predictors.

As patients could perform a maximal effort in some FCE tests and be self-limited in others, we divided the sample in two groups for descriptive purpose (Table 1) as follows: maximal effort in each FCE test versus at least one self-limited.

**Table 1:** Summary statistics

Type of variable	Variable	N	Possible values	Descriptive statistics		
				Entire cohort (N = 298)	Safe maximal effort (N = 153)	Self-limited effort (N = 145)
Biological	Age	298		42.52 (± 11.44)	41.48 (± 11.55)	43.61 (± 11.26)
	Gender	298	Female	8 (2.7%)	6 (3.9%)	2 (1.4%)
			Male	290 (97.3%)	147 (96.1%)	143 (98.6%)
	BMI	296		28.46 (± 4.47)	28.20 (± 4.42)	28.74 (± 4.53)
	Injuries location	298	Upper limb	133 (44.6%)	46 (30.1%)	87 (60%)
			Lower limb	111 (37.3%)	78 (51%)	33 (22.8%)*
			Spinal	43 (14.4%)	21 (13.7%)	22 (15.1%)
			Multiple site	11 (3.7%)	8 (5.2%)	3 (2.1%)
	AIS	292	Minor	102 (34.9%)	34 (22.8%)	68 (47.6%)*
			Moderate/serious	190 (65.1%)	115 (77.2%)	75 (52.4%)
	Interval between injury and hospitalization	292		562.70 (± 531.58)	553.17 (± 508.13)	572.76 (± 556.91)
	BPI severity subscale	255	0–10	4.36 (± 1.77)	3.91 (± 1.72)	4.90 (± 1.68)*
Social	Native language	296	French	102 (34.5%)	71 (46.4%)	31 (21.7%)
			Other	194 (65.5%)	82 (53.6%)	112 (78.3%)*
	Education level	298	High	113 (37.9%)	61 (39.9%)	52 (35.9%)
			Low	185 (62.1%)	92 (60.1%)	93 (64.1%)
	Employment contract	296	Yes	136 (46.0%)	76 (50.0%)	60 (41.7%)
			No	160 (54.0%)	76 (50.0%)	84 (58.3%)
	Work related injury	294	Yes	183 (62.2%)	85 (56.7%)	98 (68.1%)*
			No	111 (37.8%)	65 (43.3%)	46 (31.9%)
Psychological FAM components	TSK	269	17–68	46.30 (± 7.66)	45.55 (± 7.54)	47.16 (± 7.73)
	PCS	256	0–52	24.38 (± 12.11)	21.76 (± 11.16)	27.54 (± 12.50)*
	HAD-D	269	0–21	7.35 (± 4.00)	6.82 (± 3.90)	7.96 (± 4.03)*
Predictors	CPFI	254	0	52 (20.5%)	35 (25.0%)	17 (14.9%)
			1	46 (18.1%)	29 (20.7%)	17 (14.9%)*
			2	77 (30.3%)	41 (29.3%)	36 (31.6%)
			3	79 (31.1%)	35 (25.0%)	44 (38.6%)
	Disability (HFS/SFS)	276	0–200	115.53 (± 46.76)	130.20 (± 44.48)	99.07 (± 43.84)*
Outcomes	FCE floor-to-waist lift	298	0–50	20.12 (± 10.67)	25.07 (± 9.05)	14.90 (± 9.73)*
	FCE waist-to-overhead lift	281	0–30	13.61 (± 6.59)	16.41 (± 5.51)	10.37 (± 6.25)*
	FCE carrying dominant-hand	283	0–50	18.41 (± 9.21)	21.04 (± 7.88)	15.36 (± 9.73)*

*N* available data for each variable, possible values—range for continuous variables and categories for dichotomized variables, descriptive statistics—mean value (± standard deviation) for continuous variables or absolute number (relative number) for binary or categorical variables, age in years, *BMI* body mass index (kg/m^2^), *AIS*—abbreviated injury scale, interval between injury and hospitalization in days, *BPI* brief pain inventory, *TSK* Tampa Scale for Kinesiophobia, *PCS* pain catastrophizing scale, *HAD-D* hospital anxiety and depression scale, depression subscale, *HFS* hand function sort, *SFS* spinal function sort, normalized to 200 points, *FCE* functional capacity evaluation (kg). *Indicates a significant difference (p < 0.05) between safe-maximal and self-limited effort group

All analyses were performed using Stata 13.1 (StataCorp, College Station, Texas, USA). The significance level was set as a probability less than 0.05.

## Results

The sample characteristics are detailed in Table 1. The 298 eligible patients during the study period were included, without loss. Patients were predominantly middle-aged men (mean of 42.52 years, 97.3% of men), more than half of them did not speak French as their native language and had a low level of education. Injury locations were as follows: 44.6% upper limb, 37.3% lower limb, 14.4% spinal injury, 3.7% multiple site injury. The majority of injuries occurred at work (62%), and were classified as moderate to severe injuries according to the Abbreviated Injury Score (AIS) for 65% of patients. The median duration between trauma and hospitalization was 15 months (interquartile range, 10–22 months).

For the entire cohort, mean maximal performances at the FCE tests were: 20.12 ± 10.67 kg for the floor-to-waist lift, 13.61 ± 6.59 kg for the waist-to-overhead lift and 18.41 ± 9.21 kg for the dominant-handed carrying test. Table 1 details patients’ characteristics and their scores for the different psychological questionnaires as well as their performances for the FCE tests. There was a majority of patients having a CPFI of 2 or more points, meaning having a high score in 2 or more of the questionnaires assessing psychological components of the FAM: for 20.5% of the cohort none of the psychological dimension was clinically positive, 18.1% presented a score of 1, 30.3% a score of 2 and 31.1% a score of 3. The mean score at the HFS/SFS questionnaire was 115.53 ± 46.76.

Results of the multivariable models are presented in Table 2. The CPFI and the perceived disability (HFS/SFS) were found to have a significant influence on the 3 FCE lifting tests, after adjustment for the confounding variables. For the floor-to-waist lift, a high CPFI was associated with lower performances (ß = − 1.12; p = 0.039), meaning that for each supplementary point at the CPFI a decrease of 1.12 kg of the maximal lifted weight is expected. In other words, the difference of the maximal lifted weight between patients with extreme CPFI scores is 3.4 kg. Lower HFS/SFS scores (which means higher perceived disability) were associated with lower performances for the floor-to-waist lift (ß = 0.09; p < 0.001), meaning that for each point of the HFS/SFS score a difference of 0.09 kg of the maximal lifted weight is expected. For a 20-point difference score, which could be considered as the Minimal Clinical Important Difference (unpublished results), the maximal lifted weight will differ by 1.8 kg. For the floor-to-waist lift, our model explained 39% of the variance.

**Table 2:** Multiple regression models for FCE performances

Outcome	Variable	Entire cohort (N = 298)			Safe maximal effort			Self-limited effort		
		Coefficient	CI (95%)	p	Coefficient	CI (95%)	p	Coefficient	CI (95%)	p
FCE floor-to-waist lift	CPFI	− 1.12	− 2.17; − 0.06	0.039	− 1.07	− 2.18; 0.05	0.060	− 0.48	−2.12; 1.16	0.566
	Disability (HFS/SFS)	0.09	0.06; 0.11	< 0.001	0.07	0.04; 0.10	< 0.001	0.06	0.02; 0.10	0.006
FCE waist-to-overhead lift	CPFI	− 0.88	− 1.54; − 0.20	0.011	− 0.78	− 1.53; − 0.04	0.040	− 0.62	− 1.66; 0.43	0.248
	Disability (HFS/SFS)	0.04	0.02; 0.06	< 0.001	0.03	0.01; 0.05	0.001	0.03	− 0.00; 0.05	0.056
FCE carrying dominant-hand	CPFI	− 1.21	− 2.28; − 0.14	0.027	− 0.62	− 1.77; 0.53	0.289	− 0.96	− 2.61; 0.69	0.254
	Disability (HFS/SFS)	0.06	0.04; 0.09	< 0.001	0.04	0.01; 0.07	0.005	0.04	0.00; 0.09	0.039

*CPFI* cumulative psychological factor index, *HFS* hand function sort, *SFS* spinal function sort, Each association is adjusted for confounding variables: age, BMI, gender, trauma location, AIS, duration between injury and hospitalization, BPI severity subscale, native language, high education, employment contract, work related injury

For the waist-to-overhead lift similar associations were found. A high CPFI was associated with lower performances (ß = − 0.88; p = 0.011). A low HFS/SFS score was associated with lower performance at the waist-to-overhead lift (ß = 0.04; p < 0.001). For the waist-to-overhead lift, our model explained 42% of the variance.

For the dominant-handed carrying test a high CPFI was associated with lower performances (ß = − 1.21; p = 0.027). A low HFS/SFS score was associated with lower performances at the dominant-handed carrying test (ß = 0.06; p < 0.001). For the dominant-handed carrying test, our model explained 23% of the variance.

Table 1 details characteristics of patients having performed a safe maximal effort or a self-limited one [9]. Here we defined the two groups based on all three FCE tests, contrary to the regression models. Significant differences between groups having performed safe maximal or self-limited effort were detected for the following variables: in the self-limited effort group, we found a higher proportion of patients of foreign native language, having had work-related injury, mainly affecting upper limb, with minor severity trauma. They also show higher subjective scores for pain severity, catastrophism and depressive symptoms. However, except for age, no other confounding variable was associated with FCE results in multiple regression. Patients with self-limited efforts differed on the level of maximal performance accomplished as we found the following differences for the 3 lifting tests: 10.17 kg for the floor-to-waist lift, 6.04 kg for the waist-to-overhead lift and 5.68 kg for the dominant-handed carrying test. No associations between the CPFI and the FCE lifting tests were found among patients with self-limited efforts (Table 2). However, a low HFS/SFS score was associated with lower performances in the floor-to-waist lift (ß = 0.06; p = 0.006) and dominant-handed carrying test (ß = 0.04; p = 0.039) (Table 2). The HFS/SFS score was also marginally associated with the waist-to-overhead test (ß = 0.03; p = 0.056). Among patients performing a safe maximal effort, the CPFI was significantly associated with performances for the waist-to-overhead lift (ß = − 0.78; p = 0.040) and marginally with the floor-to-waist lift (ß = − 1.07; p = 0.060). The CPFI was not associated with performances for the dominant-handed carrying test (ß = − 0.62; p = 0.289). The HFS/SFS score was consistently correlated with the three outcomes, where lower scores predicted lower performances. By adding the binary variable (safe maximal versus self-limited effort) and its interaction with the CPFI and the HFS/SFS risk factors in our model, the explained variance was slightly greater: 52% (floor-to-waist), 49% (waist-to-overhead), and 34% (dominant-handed carrying test) respectively.

As a sensitivity analysis, we ran the same regression models but on complete-case data only (N = 212) (see Table, Online Appendix which presents the results of the regression models in complete-case data analysis). Associations between FCE tests and the CPFI were very similar to those obtained with the multiple imputation models but complete-case analysis would suggest slightly stronger relationships. Associations between FCE tests and the HFS/SFS score were the same as observed with multiple imputation data.

## Discussion

According to our first hypothesis, after adjusting with numerous confounding variables, our results show that patients with a higher number of risk factors will produce a lower performance in lifting tasks. Indeed, patients with a high CPFI were less likely to perform well at the different FCE lifting tests. The expected difference in the maximal lifted weight between a patient with a score of 3 on the CPFI and a patient with a score of 0 is 3.4 kg for the floor-to-waist test, 2.6 kg for the waist-to-overhead test and 3.6 kg for the dominant-handed carrying test. These differences may seem to be small; however, when taking into consideration the last step of the test, which is validated after five repetitions, it represents greater differences in the total cumulative handling: up to, 16.8 kg for the floor-to-waist lift and 13.2 kg for the waist-to-overhead test. Previous studies on the possible link between the FAM and FCE, focusing on the individual severity level of different risk factors, have produced mixed results [16]. The number of psychological risk factors may have a greater predictive value on outcomes than the severity level of an individual risk component [3, 17, 18]. Our results suggest a clinical interest in using the CPFI as a cumulative index when addressing FAM. This is also in accordance with other previous work assessing the cumulative risk. For example, the Start Back Tool is another cumulative tool developed to facilitate identification of low back pain patients’ level of risk of chronicity. It is based on 9 items covering physical and psychological factors. It has mainly been used as a method to detect patient at risk of chronicity, and was found to have a better predictive value on global change than single-construct questionnaires [37] or help predict subjective disability 6 months after [38]. We found no study addressing its influence on more objective physical function tests or comparing it with CPFI.

A high perceived disability was also consistently associated to poorer outcomes. This strong association may be explained by the similarity between tasks presented in the HFS/SFS questionnaires to assess perceived capacity and the objective lifting tasks performed during the FCE. This result is in accordance to previous investigations stating an influence of functional self-efficacy on FCE lifting performances [14, 15, 39]. This also suggests that the perceived disability is an important component of the FAM and must be addressed when studying the cumulative risk of the FAM on physical performance.

Contrary to our expectations and general intuition the secondary hypothesis was not confirmed. Our results showed that the CPFI was not associated with performances of patients producing a self-limited effort despite higher psychological scores. Nevertheless we found an influence for patients who achieved a safe maximal performance, a high CPFI being related with lower performances for the floor-to-waist test and the waist-to-overhead test. This result suggests the pertinence to assess the level of physical performance when studying the possible relationships between FCE results and psychological risk factors. Indeed, predictive value of FAM components may be underestimated in studies that include patients achieving self-limited efforts, which may also explain the weakness or the absence of associations between pain-related fears and FCE observed in previous studies [16]. Interestingly, no association was found between CPFI and the dominant-handed carrying test. As this activity is closer to everyday life practice when compared with the two other lifting tests, it might induce less apprehension and therefore activate the FAM at a lower level. However, this finding remains speculative as we did not assess the perceived harmfulness generated by each FCE test. In further research on FCE, the assessment of the a priori perceived harmfulness of physical testing, using for example task-specific tools such as the Fear Visual Analog Scale (FVAS) or the Photograph Series of Daily Activities (PHODA) would be interesting to clarify this hypothesis [40, 41]. The perceived disability (HFS/SFS) was significantly associated with each FCE test independently from the level of performance. With self-limited effort, the perceived disability was the unique FAM component influencing performances. Once again, the similarity between some items of the pictorial HFS/SFS questionnaires and the lifting tasks may be a contributing factor explaining this finding.

Sensitivity analysis showed that the associations found in the present study were not mediated by the multiple imputations method. We only observed that associations seem stronger between the FCE tests and the CPFI in complete-case analyses. Non responders were previously shown to have a higher biopsychosocial complexity [33]. Indeed these patients present adverse contextual or personal factors such as treatment resistance or impairment of social integration [42] that have not been included in our model and might therefore influence results.

This study has some limitations. First, the unusual setting with a sample mainly composed of men coming for late rehabilitation may induce a limitation for the generalization of the results. It has been well described that interval between injury and rehabilitation may influence outcome [43, 44]. After a long period of disability, related for instance with differences in national insurance systems, patients might develop behavioral strategies to protect themselves. However, it is far beyond the scope of this study to assess this particular aspect that could be an interesting topic for future research. Nevertheless, deep psychological mechanisms in chronic pain patients are reasonably comparable and our findings are in line with a body of research suggesting the importance of the FAM [1–3, 18]. When considering the explained variance, other possible influential factors are missing in our model. Among these, motivational and volitional aspects might be the most significant [1, 3]. Other personal and contextual factors, such as health literacy [45] or work-related factors [46] for instance, in the meaning of the International Classification of Functioning [47], should also be taken into consideration. Another limitation may be the use of a slightly modified CPFI which differs in two aspects from the original assessment method proposed by Wideman et al. [20]. The main modification was the use of the HADS, another standard reference questionnaire assessing depression, instead of the Beck Depression Inventory. Both instruments have high sensitivity and specificity for the diagnosis of depression [48]. The second modification was the use of a higher TSK clinical cut-off, as proposed by Roelofs [27], which permitted a better selection of patients with elevated kinesiophobia. For this reason, we believe that this did not have an important effect on our model. Due to the calculation mode of the CPFI, all variables included are treated with the same importance. This does not allow us to assess if one or the other FAM-relevant variable has a predominant impact on FCE performances. Furthermore, our relatively small sample size did not allow us to strongly establish the associations between the FAM and FCE tests in patients with a self-limited effort, as one-third of the patients completed this level of performance. Finally, the type of analysis conducted in this study allows us to state an influence of the FAM on FCE performances but does not enable us to establish a cause-effect relationship.

## Conclusion

Our study is the first investigating the cumulative influence of all the components of the FAM (catastrophizing, fear of movement, depressive symptoms and perceived disability), regarded as risk factors when above the clinical cut-off-scores, on three standard FCE lifting tasks (floor-to-waist and waist-to-overhead liftings, and carrying with dominant hand). The strength of this study is the investigation of the cumulative impact of the multidimensional FAM on physical tasks commonly used in working or daily life areas. Our results suggest that assessors and clinicians should take into account the FAM components with a cumulative perspective when interpreting maximal physical performance during FCE. Moreover, this study highlighted the likely influence of a maximal versus a self-limited effort when studying relationships between psychological factors and objective physical tasks.

Our results do not imply causal relationships between FAM and physical performance during lifting tasks. RCTs would be needed to measure effects of therapeutic interventions on physical performance and fear-beliefs during FCE, for instance graded exposure and/or cognitive behavioral therapy [49]. In a vocational rehabilitation perspective, longitudinal studies would also be needed to analyse the effect on return to work.

## Electronic supplementary material

Below is the link to the electronic supplementary material.

Online Appendix (DOCX 16 KB)
