# Supplementary material for: Predictive Value of the Fear-Avoidance Model on Functional Capacity Evaluation
Source: J Occup Rehabil. 2017 Nov 1;28(3):513–22. doi: 10.1007/s10926-017-9737-7 (PMC6096494; doi:10.1007/s10926-017-9737-7)
Supplement: Supplementary file 1 — Online Appendix (DOCX 16 KB) [file 10926_2017_9737_MOESM1_ESM.docx]

**Table Appendix 1.** Results of the regression models in complete-case data analysis.

|  |  | **Entire cohort (N=212)** | | | **Safe maximal effort** | | | **Self-limited effort** | | |
| --- | --- | --- | --- | --- | --- | --- | --- | --- | --- | --- |
| **Outcome** | **Variable** | **Coefficient** | **CI (95%)** | **p** | **Coefficient** | **CI (95%)** | **p** | **Coefficient** | **CI (95%)** | **p** |
| **FCE floor-to-waist lift** | **CPFI** | -1.25 | -2.48 ; -0.01 | 0.049 | -1.36 | -2.61 ; -0.11 | 0.033 | -0.76 | -2.88 ; 1.36 | 0.482 |
|  | **Disability (HFS/SFS)** | 0.08 | 0.04 ; 0.11 | < 0.001 | 0.06 | 0.03 ; 0.09 | 0.001 | 0.05 | -0.00 ; 0.10 | 0.076 |
| **FCE waist-to-overhead lift** | **CPFI** | -1.01 | -1.76 ; -0.26 | 0.009 | -0.87 | -1.66 ; -0.09 | 0.030 | -1.00 | -2.17 ; 0.17 | 0.093 |
|  | **Disability (HFS/SFS)** | 0.04 | 0.02 ; 0.06 | < 0.001 | 0.03 | 0.01 ; 0.06 | 0.002 | 0.02 | -0.01 ; 0.05 | 0.143 |
| **FCE carrying dominant-hand** | **CPFI** | -1.10 | -2.31 ; 0.12 | 0.076 | -0.66 | -1.94 ; 0.62 | 0.312 | -1.16 | -3.06 ; 0.74 | 0.231 |
|  | **Disability (HFS/SFS)** | 0.06 | 0.03 ; 0.09 | < 0.001 | 0.04 | 0.01 ; 0.08 | 0.013 | 0.04 | -0.01 ; 0.09 | 0.097 |

CPFI = Cumulative Psychological Factor Index ; HFS = Hand Function Sort ; SFS = Spinal Function Sort ; Each association is adjusted for confounding variables : age, BMI, gender, trauma location, AIS, duration between injury and hospitalization, BPI severity subscale, native language, high education, employment contract, work related injury.
